# Sense of coherence and attrition during four-year follow-up in cohorts of permanent and non-permanent Finnish employees

**DOI:** 10.1186/1471-2458-8-88

**Published:** 2008-03-13

**Authors:** Virpi Liukkonen, Pekka Virtanen, Mika Kivimäki, Jaana Pentti, Jussi Vahtera

**Affiliations:** 1 Tampere School of Public Health, FIN-33014 University of Tampere, Finland; 2 Department of Epidemiology and Public Health, University College London, 1-19 Torrington Place, London, WC1E 6BT, UK; 3 Finnish Institute of Occupational Health, Hämeenkatu 10, FIN-20500 Turku, Finland

## Abstract

**Background:**

We studied whether health resources, measured as sense of coherence (SOC), are associated with participation in a follow-up survey among permanent and non-permanent employees who responded at baseline.

**Methods:**

Of a cohort of 5,981 permanent employees, those who after four years were still in the service of the same employer were asked to participate in a follow-up survey. Another cohort consisted of 2,194 fixed-term and 682 subsidised employees; among these the follow-up survey was posted to those whose addresses were found in the population register. Non-participation was divided into loss to follow-up (i.e., failure to locate the individual, death and, among permanent employees, turnover or exit from labour market) and non-response to the follow-up survey. Logistic regression analyses were used to examine whether the respondents differed from the non-respondents with respect to SOC and other characteristics at baseline.

**Results:**

Among permanent employees the follow-up survey yielded 3,998 respondents, 1,051 were lost, and 932 did not reply. Among non-permanent employees the follow-up survey yielded 1,563 respondents on initially fixed-term and 467 on subsidised contracts, the corresponding figures for those lost were 145 and 38, and for the non-respondents 486 and 177. Low SOC was associated with lower response rate among fixed-term but not among permanent or subsidised employees. No association was found between SOC and loss to follow-up.

**Conclusion:**

SOC is a potential source of non-random sample attrition and should be taken into account for when estimating bias due to non-participation in occupational cohorts that include fixed-term employees.

## Background

The epidemiological knowledge of work-related risks is based mostly on follow-up studies of occupational cohorts in particular workplaces. Such studies are becoming more complicated, as the instability of work life is increasing due to increasing proportions of various temporary job contracts and higher employee turnover between organisations and occupations. In addition to focusing on the cohort that remains in stable employment during the follow up, epidemiological studies should also trace those employees who left their workplaces, and control for bias related to such attrition.

Sample attrition and the accompanying bias impair the interpretation of the results of health surveys. The reasons for attrition are, in addition to refusal to participate, death or severe illness and failure to locate the individuals. Distinguishing those who cannot be contacted is important, because the mechanism of non-participation may influence the nature of the attrition bias [1-3]. In a cohort study within a workplace or organisation one notable reason for attrition is turnover of workers, which includes many sub-categories (e.g. transfer to other workplaces, layoffs and outsourcings, retirement). Instead, in a study of the cohort also including employees who leave the organisation, difficulties in finding individuals' place of residence and address are important reason for attrition.

To the best of our knowledge, only a few longitudinal population-based studies have taken into account sample attrition related to the working situation. The employed tend to participate more actively than unemployed [1,4,5]. Badawi et al. [6] found that the unemployed were more difficult to trace and died more often, but unemployment did not influence refusal to participate. The traditional dichotomy of unemployed versus employed, however, is too crude for assessing the modern labour market, where employees may occupy several statuses between permanent jobs and overt unemployment. For example, there is no research among fixed-term employees.

The existing research on the determinants of attrition focuses on sociodemographic factors and health, including mental disorders, but psychological characteristics that may also predict sample attrition are rarely included in these studies. We were interested in the sense of coherence (SOC) [7,8], as a possible source of bias in a cohort study. According to Antonovsky's theory of SOC, a person with strong SOC stays healthy despite possible stressful encounters and a number of empirical studies show that strong SOC is indeed associated with various aspects of good health [9-13]. SOC-related non-response might be hypothesised to be as a potential source of bias in health-related survey studies, but there is no research available on a possible association of SOC with attrition or participation.

The aim of this study was to examine sample attrition in a four-year follow-up study among cohorts with initially permanent and non-permanent employment status. We examined baseline characteristics between employees who participated in a follow-up study compared with those who did not. Non-participation was categorised into drop-out due to loss and drop-out due to refusal. Our particular aim was to investigate SOC as a possible source of bias, i.e. we asked whether low SOC predicts refusal to participate in a follow-up survey or exit from the work place or disappearance from the population register. Moreover, we aimed to investigate the associations of participation with sociodemographic characteristics and health in the cohorts of permanent and non-permanent employees.

## Methods

### Participants

This study was carried out in eight Finnish towns involved in two cohort studies, the 10-Town Study and the Temporaries in Municipal Jobs Study (TMJ Study), both investigating the relationships between behavioural and psychosocial factors and health among municipal employees. The ethics committee of the Finnish Institute of Occupational Health approved the study.

In 1997 the 10-Town Study distributed the initial questionnaire to the workplaces of the participants – full-time employees who were employed by the town. In the small towns the entire municipal personnel was included in the study, while in the three largest towns, a random sample from each town was used. A total of 6,442 employees replied to the initial survey, giving a response rate of 67%. After exclusion of respondents found to be on a non-permanent contract (n = 461), the sample included 5,981 permanent employees. Those who were still employed in the initial municipality were asked to participate in a follow-up survey in 2001.

The TMJ Study was carried out among all employees of the same towns who had a non-permanent job contract in November 1997. This survey (with a response rate of 57%) yielded 2,194 participants who reported having a fixed-term contract (i.e. their contract expired at a predetermined point in time because of substitution or "for some other reason"), and 682 subsidised employees (i.e. men and women who had been unemployed for more than 12 months and who were now on a 6 to10-month work contract funded through a government programme to support and enhance the working ability of the long-term unemployed). The follow-up survey was posted in 2002 to all those whose addresses were found in the Finnish Population Register.

### Sense of coherence

SOC was assessed using the 13-item version of the Orientation to Life Questionnaire devised by Antonovsky [8,14]. The questions ask the respondents to check their level of agreement with items on a seven-point semantic differential scale with two anchoring phrases. In the analysis the respondents were divided into three groups (the highest quartile, the two middle quartiles, the lowest quartile) on the basis of their mean SOC.

### Demographic variables and health at baseline

Data on the respondents' occupations were derived from employers' records, and four categories were defined on the basis of the International Standard Classification of Occupations [15]: professionals (ISCO titles 1–2), associate professionals (title 3), clerks (title 4) and manual workers (titles 5–9). Age was categorised into three groups: 17 to 29-year-olds, 30 to 49-year-olds and 50 to 64-year-olds.

Self-rated health was the respondents' overall assessment of their health and was measured with a single question offering five options and dichotomised into good (excellent or fairly good) and poor (average, fairly poor or poor).

### Statistical analysis

We carried out logistic regression analyses to obtain the odds ratios and their 95 percent confidence intervals that reflected the associations between the SOC, demographic characteristics and self-rated health and the two types of attrition. The analyses were adjusted for age, sex, occupational status and baseline level of self-rated health. The respondents of the follow-up survey served as the comparison category. SPSS for Windows version 13.0 statistical software was used in the analyses.

## Results

### The cohort of permanent employees

Among the participants of the baseline survey, 76 per cent of the permanent employees were women, the mean age was 45 years, and 33 per cent reported poor self-rated health (Table 1). The follow-up survey was distributed to 4,930 (82 per cent of the baseline participants). The baseline respondents who were not eligible at follow-up (n = 1,051, 18 per cent of the baseline participants) consisted of those who had died or had retired because of age or illness and those who had moved to another workplace. The follow-up survey yielded 3,998 respondents (response rate 81 per cent) while 932 employees refused to participate.

**Table 1:** Characteristics of permanent employees who responded in the baseline and follow-up surveys and who did not respond in the follow-up survey, and odds ratios and 95% confidence intervals for response at follow-up by characteristic.

	Participants of baseline survey n = 5981	Respondents of follow-up survey n = 3998	Non-respondents to follow-up survey			
			
			Lost^a ^(n = 1051)		Refused (n = 932)	
			
	(%)	(%)	(%)	OR (95% Cl)^b^	(%)	OR (95% Cl)^b^
Sense of coherence						
High	23	23	23	1	23	1
Intermediate	52	52	50	1.02 (0.85–1.22)	50	1.08 (0.89–1.30)
Low	25	25	27	0.94 (0.76–1.16)	27	0.99 (0.79–1.23)
Gender						
Men	24	22	22	1	33	1
Women	76	78	78	0.84 (0.70–1.01)	67	**1.58 (1.34–1.86**)
Age group						
17 – 29	3	2	6	1	5	1
30–49	62	66	41	**5.45 (3.81–7.78)**	66	**2.23 (1.50–3.31)**
50–64	35	32	53	**2.15 (1.50–3.08)**	29	**2.51 (1.67–3.78)**
Occupational status						
Professionals	38	37	44	1	36	1
Associate professionals	21	23	17	**1.69 (1.38–2.07)**	18	**1.25 (1.01–1.54)**
Clerks	9	10	8	**1.54 (1.18–2.02)**	7	1.27 (0.94–1.71)
Manual workers	32	30	31	**1.37 (1.15–1.63)**	39	**0.79 (0.66–0.94)**
Self-rated health						
Good	67	70	59	1	66	1
Poor	33	30	41	**0.66 (0.56–0.77)**	34	**0.82 (0.69–0.96)**

^a ^Employees no longer were in the service of the same employer as at baseline

^b ^Adjusted for age, sex, occupational status and baseline level of self-rated health

Exit from the follow-up cohort of permanent employees did not depend on SOC (Table 1). Exit was most probable in the youngest and in the professionals. Poor self-rated health also predicted exit.

Non-response at follow-up was not associated with SOC (Table 1). Participation was less likely among men, in the youngest and in the manual workers. Moreover, non-respondents had more likely poor self-rated health.

### The cohort of fixed-term and subsidised employees

At baseline, 82 per cent of the fixed-term employees were women, the mean age was 35 years, and 17 per cent reported poor self-rated health (Table 2). The corresponding figures for the subsidised employees were 77 per cent, 38 years and 31 per cent (Table 3). The proportion of manual jobs was higher among the subsidised employees than in other employment types.

**Table 2:** Characteristics of fixed-term employees who responded in the baseline and follow-up surveys and who did not respond in the follow-up survey, and odds ratios and 95% confidence intervals for response at follow-up by the characteristic.

	Participants of baseline survey (n = 2194)	Respondents of follow-up survey (n = 1563)	Non-respondents to follow-up survey			
			
			Lost^a ^(n = 145)		Refused (n = 486)	
			
	(%)	(%)	(%)	OR (95% Cl)^b^	(%)	OR (95% Cl)^b^
Sense of coherence						
High	30	31	26	1	28	1
Intermediate	49	50	46	1.02 (0.66–1.57)	48	0.93 (0.72–1.21)
Low	21	19	28	0.68 (0.41–1.14)	24	**0.69 (0.50–0.94)**
Gender						
Men	18	16	17	1	25	1
Women	82	84	83	1.02 (0.63–1.66)	75	**1.68 (1.29–2.19)**
Age group						
17 – 29	35	33	42	1	37	1
30–49	54	55	52	1.43 (0.98–2.09)	54	1.07 (0.85–1.36)
50–64	11	12	6	**2.43 (1.16–5.09)**	9	**1.52 (1.02–2.27)**
Occupational status						
Professionals	42	42	53	1	39	1
Associate professionals	22	22	14	**2.06(1.27–3.50)**	23	0.87 (0.66–1.16)
Clerks	10	10	9	1.29 (0.68–2.41)	10	0.99 (0.68–1.48)
Manual workers	26	26	24	1.40 (0.89–2.17)	28	0.82 (0.62–1.07)
Self-rated health						
Good	83	83	79	1	84	1
Poor	17	17	21	0.75 (0.47–1.19)	16	1.20 (0.88–1.63)

^a ^Among fixed-term employees those whose addresses were not found in the population register

^b ^Adjusted for age, sex, occupational status and baseline level of self-rated health

**Table 3:** Characteristics of subsidised employees who responded in the baseline and follow-up surveys and who did not respond in the follow-up survey, odds ratios and 95% confidence intervals for response at follow-up by characteristic.

	Participants of baseline survey (n = 682)	Respondents of follow-up survey (n = 467)	Non-respondents to follow-up survey			
			
			Lost^a ^(n = 38)		Refused (n = 177)	
			
	(%)	(%)	(%)	OR (95% Cl)^b^	(%)	OR (95% Cl)^b^
Sense of coherence						
High	20	21	16	1	20	1
Intermediate	47	48	30	1.16 (0.38–3.56)	46	1.10 (0.67–1.83)
Low	33	31	54	0.37 (0.13–1.10)	34	0.94 (0.54–1.63)
Gender						
Men	23	21	26	1	28	1
Women	77	79	74	1.03 (0.44–2.46)	72	**1.63 (1.04–2.54)**
Age group						
17 – 29	29	29	29	1	29	1
30–49	48	45	55	0.77 (0.32–1.84)	55	0.83 (0.53–1.30)
50–64	23	26	16	1.52 (0.49–4.76)	16	**1.92 (1.06–3.48)**
Occupational status						
Professionals	8	10	3	1	6	1
Associate professionals	15	15	15	0.28 (0.03–2.55)	15	0.59 (0.25–1.43)
Clerks	27	27	15	0.63 (0.07–5.63)	26	0.61 (0.27–1.38)
Manual workers	50	48	67	0.23 (0.29–1.77)	53	0.55 (0.25–1.20)
Self-rated health						
Good	69	69	68	1	68	1
Poor	31	31	32	1.27 (0.54–2.97)	32	0.99 (0.64–1.54)

^a ^Among subsidised employees those whose addresses were not found in the population register

^b ^Adjusted for age, sex, occupational status and baseline level of self-rated health

The follow-up survey was posted to 2,049 (93 per cent) fixed-term and 644 (94 per cent) subsidised respondents of the initial survey whose addresses were found in the population register. Among fixed-term employees the follow-up survey yielded 1,563 respondents (response rate 76 per cent) while 486 refused to participate. Among subsidised employees the corresponding figures were the 467 (response rate 73 per cent) and 177.

Crude figures suggested that loss from the cohorts was dependent on SOC, but the odds ratios showed the associations were statistically non-significant both among the fixed-term and in among subsidised employees (Tables 2 and 3). Among the fixed-term employees loss was least common in the oldest and among associate professionals; among subsidised employees no significant associations with the studied variables were seen.

Non-response at follow-up was associated with low SOC among the fixed-term employees (Table 2) but not among the subsidised employees (Table 3). In both cohorts, women and the oldest individuals were more likely to participate.

## Discussion

This study examined sample attrition in cohorts of permanent and non-permanent employees. The focus was on the associations of baseline SOC with non-response to follow-up survey. In addition, we examined whether SOC predicted contact failure due to exit from the work place among permanent employees and contact failure due to unlocated address among fixed-term and subsidised employees. Corresponding associations with sociodemographic factors and self-rated health were also studied.

We found that low SOC predicted low response rate in the cohort of fixed-term employees. Contact failure was not related to SOC among permanent employees, i.e. those who left work for "natural" reasons such as a move to another workplace, retirement or death, had only slightly lower SOC than the respondents. Among non-permanent employees contact failure tended to be more common in those with low SOC, but this association did not reach statistical significance.

According to Antonovsky [7,8] SOC predicts and explains an individual's movement along the health-disease continuum. He viewed SOC as individuals' global orientation or disposition leading to a certain attitude towards their internal and external environment [8]. A strong SOC means good resources to resist stressful encounters, including work-related stress and other exposures. In line with this, Feldt [16] found that employees with strong SOC had fewer psychosomatic symptoms and lower level of emotional exhaustion at work. Antonovsky's theory also suggests that SOC stabilises by the age of about 30, and the first years of employment are important in this development [8]. The period during which individuals leave their studies to enter the labour market appears particularly important [17]; if the early career is characterised by insecure entry into work, SOC may never reach the optimal level.

In our study, the permanent employees were older than the fixed-term and subsidised employees, and it is evident that majority of them should have had a stable SOC. However, as most of the non-permanent employees were older than 30, and we may assume that their SOC was also relatively stable.

Research in the field of psychiatry has documented that psychiatric disorders and psychopathology increase sample attrition due to loss of contact rather than refusal [6,18]. According to our results SOC does not follow this tendency. In other words it seems to be inappropriate to consider SOC simply as an indicator of mental morbidity or psychological well-being.

Poor self-rated health has been shown to predict sample attrition [1,5,19]. Our findings confirm this in the cohort of permanent employees, both with regard to non-response and to exit. In fixed-term employees the point estimate showed an opposite though statistically non-significant association with regard to non-response. On the other hand, among them responding was associated with high SOC. In all, these findings add one more paradox to the body of research concerning health and well-being of fixed-term employees [see e.g [20]].

Women tend to participate in follow-up more actively than men [19,21-23], although there are some conflicting findings [2,24]. In this study women also were more likely to respond in all cohorts. Several studies have explained this by pointing to gender differences in health. However, in our study such an explanation was unlikely because the analyses were adjusted for self-rated health at baseline.

The probability of responding is reported in most studies to increase with age [7,10,12], although there are studies of elderly cohorts reporting opposite findings [3,12]. In our study the cohorts were relatively young. Thus, our finding of less attrition among older participants was to be expected.

Higher response rates have been reported among those with a higher socio-economic status [1-3,6,18,19,21-24]. This finding was replicated in our study but only among permanent employees.

This study, concentrating on associations between SOC and sample attrition during follow-up, does not permit conclusions of a potential SOC-related participation bias in the initial survey. It is possible that the effect of SOC is different in surveys conducted among unselected populations.

## Conclusion

In the past, it was easier to follow up occupational cohorts throughout their working careers, as it was common for individuals to stay in one workplace. Nowadays such follow-up is difficult due to greater turnover in occupations and employers and employees. This study showed that it is possible to trace over 90 per cent of non-permanent employees by utilizing the Finnish Population Register. Inclusion of non-permanent employees, however requires, special arrangements, and among them sample attrition may be related to different factors than among permanent employees. Our finding of non-random sample attrition in relation to SOC serves as an example of factors which must be considered when following up occupational cohorts that include fixed-term employees.

## Abbreviations

SOC: Sense of Coherence.

## Competing interests

The author(s) declare that they have no competing interests.

## Authors' contributions

VL carried out the statistical analyses and was responsible for writing the drafts of the manuscript. PV participated in designing the study and in the statistical analyses and contributed to the writing of the manuscript. JP was responsible for production of the survey and register data and for the construction of the variables. MK and JV participated in designing the study and contributed to the writing of the manuscript. All authors read and approved the final manuscript.

## Pre-publication history

The pre-publication history for this paper can be accessed here:


